# Reaching Absent and Refusing Individuals During Home-Based HIV Testing Through Self-Testing—at What Cost?

**DOI:** 10.3389/fmed.2021.653677

**Published:** 2021-06-29

**Authors:** Alain Amstutz, Lineo Matsela, Thabo Ishmael Lejone, Mathebe Kopo, Tracy Renée Glass, Niklaus Daniel Labhardt

**Affiliations:** ^1^Clinical Research Unit, Department of Medicine, Swiss Tropical and Public Health Institute, Basel, Switzerland; ^2^University of Basel, Basel, Switzerland; ^3^Department of Infectious Diseases and Hospital Epidemiology, University Hospital Basel, Basel, Switzerland; ^4^Health Economics Unit, Faculty of Health Sciences, University of Cape Town, Cape Town, South Africa; ^5^SolidarMed, Partnerships for Health, Butha-Buthe, Lesotho

**Keywords:** human immunodeficiency virus, self-testing, secondary distribution, Lesotho, Southern Africa, cluster-randomized trial, cost analysis

## Abstract

**Introduction:** In the HOSENG trial (NCT03598686), the secondary distribution of oral self-tests for persons absent or refusing to test during a home-based HIV testing campaign in rural Lesotho resulted in an increase in testing coverage of 21% compared to a testing campaign without secondary distribution. This study aims to determine the per patient costs of both HOSENG trial arms.

**Method:** We conducted a micro-costing study to estimate the cost of home-based HIV testing with (HOSENG intervention arm) and without (HOSENG control arm) secondary self-test distribution from a provider's perspective. A mixture of top-down and bottom-up costing was used. We estimated both the financial and economic per patient costs of each possible testing cascade scenario. The costs were adjusted to 2018 US$.

**Results:** The overall provider cost for delivering the home-based HIV testing with secondary distribution was US$36,481 among the 4,174 persons enumerated and 3,094 eligible for testing in the intervention villages compared to US$28,620 for 3,642 persons enumerated and 2,727 eligible for testing in the control. The cost per person eligible for testing was US$11.79 in the intervention vs. US$10.50 in the control. This difference was mainly driven by the cost of distributed oral self-tests. The cost per person tested was, however, lower in intervention villages (US$15.70 vs. US$22.15) due to the higher testing coverage achieved through self-test distribution. The cost per person confirmed new HIV+ was US$889.79 in the intervention and US$753.17 in the control.

**Conclusion:** During home-based HIV testing in Lesotho, the secondary distribution of self-tests for persons absent or refusing to test during the visit reduced the costs per person tested and thus presents a promising add-on for such campaigns.

**Trial Registration:**https://ClinicalTrials.gov/, identifier: NCT03598686

## Introduction

In 2019, 87% of all people living with HIV in eastern and southern Africa were aware of their status. However, 530,000 people still remained undiagnosed and may be hard to reach through traditional HIV testing services (1). Door-to-door HIV testing campaigns in southern Africa have the potential to increase early diagnosis, reach people that rarely use traditional health services, and yield testing uptake of more than 90% (2–5). However, such testing campaigns are costly and testing coverage—the proportion of a surveyed population tested—often remains low because of absent household members during the campaign day (2, 6, 7). The World Health Organization (WHO) recommends HIV self-testing as a complement to current testing approaches, and thus HIV self-tests are also increasingly offered during door-to-door testing campaigns (8). The HOSENG (HOme-based SElf-testiNG) cluster-randomized trial in rural Lesotho assessed the effect of the one-time secondary distribution of oral-fluid self-tests to absent and household members who refuse standard blood-based HIV testing during a home-based testing campaign on testing coverage. It resulted in 21% higher testing coverage compared to no secondary distribution, however without investigating the cost implications (9).

One common approach to assess the per-patient costs of HIV services is to compare the unit costs such as cost per person tested or cost per diagnosis using either a top-down (total expenditure assigned per arm according to an allocation factor based on patient volume) or bottom-up (sum of each resource use individually calculated according to actual usage) approach (10, 11). A systematic literature review commissioned by the WHO summarized that home-based HIV testing in sub-Sahara Africa incurred a median cost of US$11 per person tested (10), from as low as US$7 in Kenya (12) to US$14 in Lesotho (5) and US$19 in Uganda (13). There are only a few home-based testing studies from the region that assess the testing coverage of the entire surveyed area, and those among them who investigated costs reported costs per person tested ranging from US$3.02 to US$20.50 (7, 14–16). None of these studies include HIV self-testing. Published costing data that evaluated the costs per HIV self-test distributed during home-based testing, including program expenditure, range from US$8.15 in Malawi (17) to US$43.30 in Lesotho (18). Costing data on secondary HIV self-test distribution during home-based testing, however, are scarce, with only one cluster-randomized trial from Zambia reporting such data. Self-tests were offered during a door-to-door campaign and distributed among absent partners of present household members. The researchers calculated that the intervention costed US$30 per person tested (19).

Based on data of the HOSENG trial in Lesotho, we report in this study the cost of home-based HIV testing with and without secondary self-test distribution assessed as the cost per person enumerated, eligible for testing, tested, and confirmed new HIV-positive. This study aims to provide scarce costing data about the secondary distribution of HIV self-tests during door-to-door testing campaigns in sub-Sahara Africa.

## Method

### The Hoseng Testing Campaign

In 2018, the HOSENG two-arm cluster-randomized trial offered home-based HIV testing in 106 village clusters in the catchment area of 20 health facilities in two rural districts in Lesotho (Butha-Buthe and Mokhotlong). The 20 health facilities serve a rural population of about 200,000 inhabitants living in a rather mountainous area with poor infrastructure. The village clusters from urban areas (e.g., Butha-Buthe town and Mokhotlong town) were excluded. A cluster was defined as a village with a consenting village chief and served by a registered and active village health worker (VHW). VHWs are the existing Lesotho Ministry of Health (MoH) lay community health worker network and are supervised by the corresponding health facility where they attend regular monthly meetings. The comprehensive details of the trial design and intervention are published elsewhere (9, 20). Briefly, a trained team of 15 campaign counselors and three drivers conducted the 5-month door-to-door testing campaign and spent 1 to 2 days per village. In both arms, the campaign team enumerated all household members living in the surveyed area and offered blood-based point-of-care HIV testing (Determine HIV-1/2 and UniGold HIV-1/2) to all household members who were present with an unknown HIV status and thus eligible for testing. Household members with a HIV-negative test within the previous 4 weeks or known to be HIV-positive were not eligible for testing.

#### Control Arm

In the 49 villages assigned to the control arm, the campaign team referred absent household members and those refusing to test to the nearby health facility. The campaign team and the provided services as outlined above were the same in both arms.

#### Intervention Arm

In the 57 intervention villages, for every household member aged 12 years or older who was absent or refused blood-based HIV testing, the team asked for consent to leave an oral-fluid HIV self-test kit (OraQuick ADVANCE HIV I/II) in the household, and one present household member was trained to correctly use the self-test. The responsible VHW, who lives and works in the same village, followed up the distributed self-tests. The VHWs from all 106 villages received a 1-day refresher training on HIV prevention, testing, counseling as well as result documentation. The VHWs from the intervention arm received additional training about oral-fluid HIV self-testing and a list of all household members for whom a self-test was dispensed. The VHWs revisited all households 2–4 weeks after the reported date of the absent family member's return to collect the self-test if it had not been returned before. The village health workers reread the result of the oral-fluid HIV self-test strip and documented the outcome on the study-specific form. The household members with a reactive self-test were referred to the clinic for blood-based testing in order to confirm an HIV-positive outcome.

#### Endpoint

After 120 days of follow-up per village, the HIV testing coverage among the enumerated population aged 12 years and older was assessed through the testing registers at all health facilities (control and intervention arm) as well as the VHWs' documentation tool (intervention arm only).

### HIV Testing and Cost Data Sources

This study included HIV testing data from all enumerated household members aged 12 years and above from HOSENG trial in both arms, including the 120-day follow-up period. The cost data were obtained through the trial expenditure records, a Lesotho Public Service Circular and Lesotho Public Health Sector Expenditure Review 2017 (21), and supplemented by interviews with the administrative staff and the study team.

### Costing Methodology

We conducted a micro-costing study to estimate the cost of home-based HIV testing with and without secondary self-test distribution from a provider's perspective. A mixture of top-down approach and bottom-up costing was used, following international guidelines (22, 23). We included both financial and economic costs, whereby financial costs reflected resources or goods that were paid for, while economic costs encompassed the valuation of donated goods and services such as the VHW and clinic counselor time provided by the MoH. Expenses only covering research activities, such as the electronic tablet-based data collection tool, were excluded.

### Unit Cost Calculation

Supplementary Table 1 provides the details of all cost inputs. The unit cost of the self-test kits was assumed to be US$2.10, which accounted for purchase and shipment. We classified the costs into independent categories: trainings, logistics, clinic overhead, campaign equipment, consumables at both facility and community level (HIV blood-based tests, oral-fluid self-tests, gloves, *etc*.), headquarter-based staff (campaign organizers), and field-/clinic-based staff (campaign counselors, MoH clinic HIV testing counselors, and MoH VHWs).

In a model constructed in Microsoft Excel®, the testing data was outlined along the possible testing scenarios that occurred in the intervention and the control arms (Supplementary Figures 1A,2B). In the same model, the field- and clinic-based staff time for each activity was determined by using a bottom-up approach, whereby the total time spent by each staff member was divided by the number of clients attended to in each scenario. For the campaign staff, this included the time spent on traveling, waiting, enumeration, and mobilization of the community. Similarly, consumable costs were determined bottom-up based on the actual number of clients per scenario. Notably, HIV self-testing costs and VHW-associated costs occurred only in the intervention scenarios. The remaining cost categories were allocated in a top-down approach and distributed equally by arm and scenario.

The costs incurred for each scenario were then summed up by arm and divided by the respective unit number achieved by arm, i.e., number of persons enumerated, number of person eligible for testing, number of persons tested, and number of persons confirmed to be HIV-positive. We also calculated the incremental cost of distributing self-tests during home-based HIV testing by subtracting the total costs of the control arm from the total costs of the intervention arm. Part of the logistics (one car provided by the research organization) and the training costs were annualized over the assumed years of useful life of each item using a 3% discount rate (23). The costs were inflated to 2018 Lesotho Loti (LSL). These were then converted to US$ using the average Central Bank of Lesotho exchange rate for 2018 (LSL 13.2517 to 1 US$).

### Sensitivity Analyses

A univariate simple sensitivity analysis was used to characterize the uncertainty in the key assumptions in the study. The impact of the discount rate was assessed by varying the rate to 0 and 5% as per Drummond et al. (22). Similarly, the years of useful life of the research organization vehicle were varied. Headquarter-based staff salaries were varied by ±10% to assess the impact of the campaign being coordinated entirely by the MoH or a higher cadre, i.e., a project nurse, as it is often the case with such campaigns. We varied the oral self-test kit price to reflect a hypothetical lower market prize to be assumed in the years to come (US$1).

### Ethics Statement

The study did not involve patient-level data collection. However, as part of the overarching HOSENG trial, we obtained permission from the Ethics Committees in Lesotho and Switzerland to extract the costing data. The HOSENG trial was approved by the National Health Research and Ethics Committee of the Ministry of Health of Lesotho (ID06-2018) and the Ethics Committee in Switzerland (Ethikkomission Nordwest- und Zentralschweiz; 2018-00283). The trial is registered under the Clinical Trials Network (ClinicalTrials.gov) under registration number NCT03598686.

## Results

There were 4,174 and 3,642 persons enumerated aged 12 years and older in the intervention and control arms, respectively. Among those, 3,094 in the intervention and 2,727 in the control were eligible for testing, as they had an unknown HIV status. In the intervention arm, 58% of the distributed self-tests were used and returned within 120 days. Overall, the intervention resulted in a significantly greater testing coverage among persons aged 12 years and above (81%) compared to the control villages (60%) in which no self-tests were dispensed. It was particularly successful among men, adolescents, and migrant workers (20).

**Figure 1:**
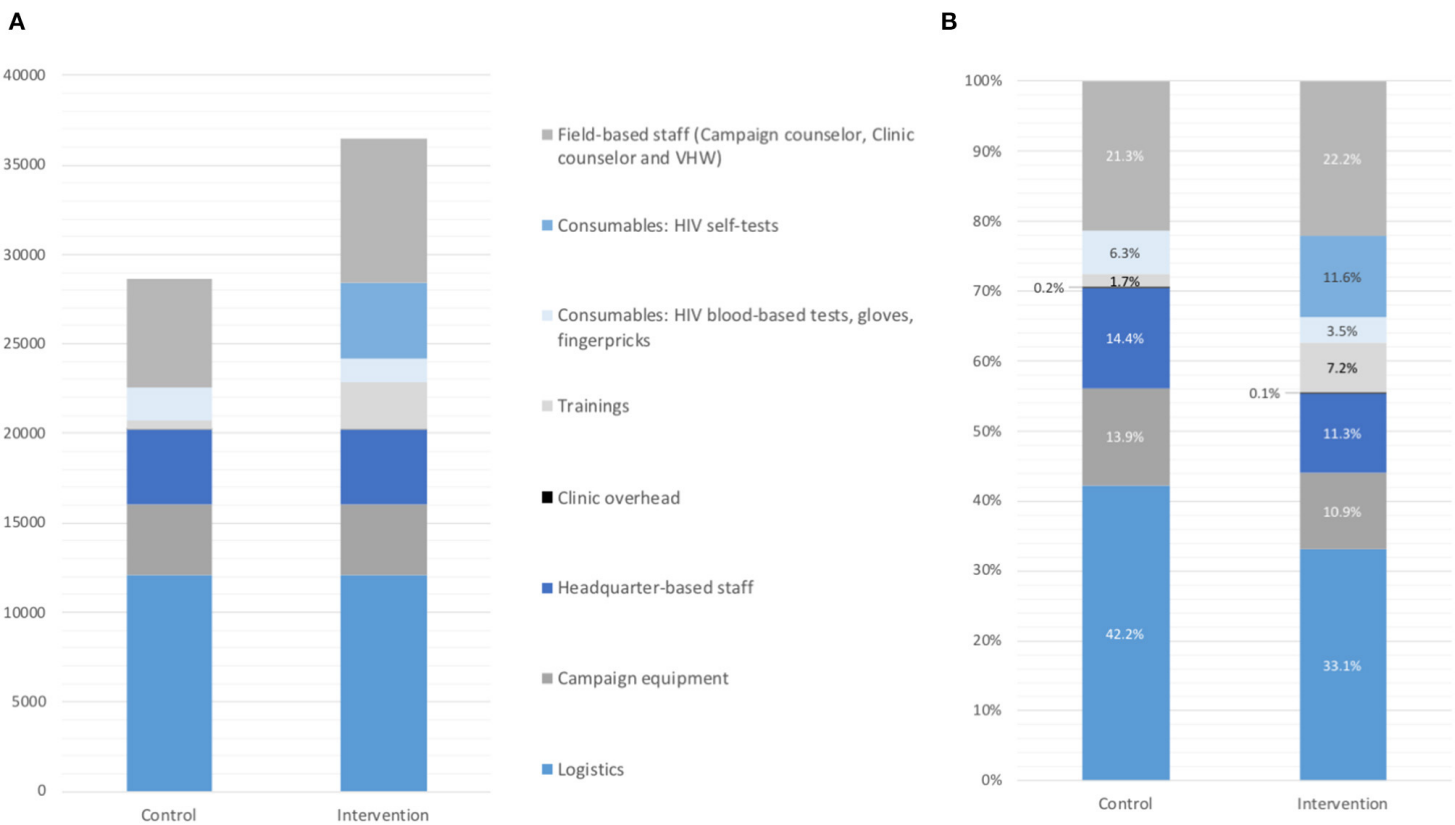
Cost item by arm in absolute US$ **(A)** and proportion of total costs **(B)**.

The overall program cost of the home-based HIV testing campaign in the control arm, where no self-tests were used nor distributed, was US$28,620. The overall program cost in the intervention arm (with secondary self-test distribution and follow-up by VHWs) was US$36,481 (Table 1). Logistics formed the largest cost item of the total costs in both arms, followed by staff costs, with the remaining costs accounting for <15% (Figure 1).

**Table 1:** Cost units by arm.

	**Intervention**	**Control**
**Cost input data (US$)**		
Logistics	12,092	12,092
Campaign equipment	3,969	3,969
Headquarter-based staff: campaign organizers	4,119	4,119
Clinic overhead	53	53
Trainings	2,622	477
Consumables	5,536	1,816
HIV blood-based tests, gloves, fingerpricks	1,287	1,816
HIV self-tests	4,248	0
Field- and clinic-based staff: campaign counselors, clinic HIV testing counselors and village health workers	8,091	6,094
Total cost	36,481	28,620
**HIV testing data (*****N*****)**		
Total number of persons enumerated ≥12years	4,174	3,642
Number of persons enumerated ≥12years, eligible for testing, with unknown HIV status	3,094	2,727
Number of persons tested	2,913	1,292
Number of persons confirmed new HIV+	41	38
Incremental number of individuals tested	1,621	
Incremental number of individuals confirmed new HIV+	3	
**Costing output (US$)**		
Cost per person enumerated	8.74	7.86
Cost per person eligible for testing	11.79	10.50
Cost per person tested	15.70	22.15
Cost per person confirmed new HIV+	889.79	753.17
Incremental costs	7,861	
Incremental cost per person tested	4.85	
Incremental cost per person confirmed new HIV+	2,620.33	

In the intervention arm, the cost per person enumerated was US$8.74, and the cost per person eligible for testing was US$11.79, whereas in the control arm, both the cost per person enumerated (US$7.86) and the cost per person eligible for testing (US$10.50) were lower (Table 1). Three cost items contributed to the higher costs in intervention (Figure 1): the oral self-tests, the additional training for the VHWs, and the field-based staff costs related to the follow-up of the distributed self-tests.

The cost per person tested, however, was lower in the intervention (US$15.70) than in the control (US$22.15), with 2,913 out of 3,094 eligible persons tested in the intervention and 1,292 out of 2,727 eligible persons tested in the control. In both arms, about 40 persons were confirmed new HIV+, resulting in unit costs per confirmed new HIV-positive person of US$889.79 in the intervention and US$753.17 in the control (Table 1).

The incremental costs of distributing and following up self-tests for absent and refusing household members alongside a home-based HIV testing campaign were estimated at US$7,861. This resulted in an incremental cost per additional person tested of US$3.38 and that of an additional person confirmed new HIV-positive of US$191.73 (Table 1).

**Figure 2:**
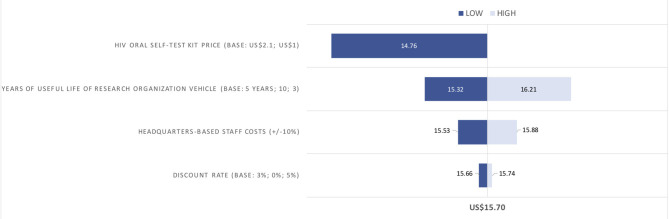
Sensitivity analyses on the costs per person tested (in 2018 US$).

The cost per person tested remained largely robust when key cost items were varied in the sensitivity analyses (Figure 2). The largest impact was observed with a lower oral self-test price, resulting in US$14.76 per person tested. Logistics accounted for the highest proportion of the total costs of the home-based testing campaign; therefore, the variation of the useful life years of the vehicle had a reasonable impact on the results (ranges from US$15.32 to US$16.21). A 10% change in headquarter-based staff salaries as well as the variation of the discount rate only had a minor effect.

## Discussion

In this costing analysis, we assessed unit costs comparing home-based HIV testing with and without secondary distribution of oral self-tests for persons absent or refusing to test during a home-based HIV testing campaign in Lesotho. The secondary distribution of oral self-tests increased the overall cost of the campaign due to the direct cost of oral self-tests and additional training cost, but due to the higher testing coverage achieved with self-tests (81%) than without (60%) and the relatively cheap follow-up of the self-tests by an existing MoH lay cadre, the secondary self-test distribution resulted in lower cost per person tested.

A previous home-based HIV testing study from Lesotho reached 72% testing coverage through catch-up visits for absent members on weekends, at a cost of US$20 per person tested (7). Using self-tests instead of catch-up visits, our intervention achieved a higher testing coverage at a lower cost per person tested (US$15.70). In Uganda and Kenya, using multi-disease community health fairs followed by home-based testing for non-attendees of the fair, 89% of all enumerated adults were reached at a cost of US$20.50 per person tested (16). Similar costs were reported in a study from Malawi, whereby a team of counselors conducted two door-to-door campaigns on Likoma, a small island in Lake Malawi, reaching a testing coverage of 89%, at US$13.50 per person tested (14). Only a study in Uganda, engaging 62 community health workers to provide regular HIV testing for 6 months, reported significantly lower costs at US$3.02 per person tested but reaching only 69% of the adult residents (15). Low travel costs as well as the involvement of community health workers (with a stipend of US$30 per month) instead of counselors probably contributed to the low costs incurred in this study.

The WHO recommends HIV self-testing to complement current testing approaches, although the cost of the most widely used self-test (OraQuick ADVANCE HIV I/II), at approximately US$2 per kit, is still around twice the price of the standard blood-based HIV rapid test in Africa (24). Thus, cost-efficient self-testing interventions to complement standard testing are needed. The Self-Testing AfRica project has delivered over 4.8 million self-tests in 38 countries through various distribution models (25). Its economic cost analysis of door-to-door community-based distribution models in Malawi, Zambia, and Zimbabwe reported average costs per self-test distributed at US$8.15, US$16.42, and US$13.84, respectively (17). A recent costing study from Lesotho calculated costs up to US$43.30 per self-test distributed when used as part of mobile outreach testing or index village testing (18). However, none of the above-mentioned community-based self-testing studies assessed testing coverage or the costs of secondary distribution.

A cluster-randomized nested trial within the HIV Prevention Trials Network 071 study in Zambia distributed self-tests among absent partners of present household members and assessed its cost implications (19). Similarly, it showed a high uptake and modestly increased the coverage from 65 to 68%. Community HIV care providers, hired by the study, performed the follow-up. In the self-testing intervention arm, the cost per person enumerated was US$18.37, and the cost per person tested was US$30, 1.37 times higher than in the control arm where no self-tests were used nor distributed. These costs were higher compared to our results, probably because of the very modest difference in testing coverage between the arms and the fact that the campaign and the follow-up were conducted by hired study staff, yielding larger personnel costs.

In a context where 81% of people living with HIV already know their status (26), the positivity yield in our study was low, with 3% during the campaign and 1% during the follow-up (20), and with only a minimal difference between the arms. A possible explanation may have been the unassisted self-testing of the secondary distributed tests and thus an underreporting of outcomes. Consequently, our cost per person confirmed new HIV+ was higher in the self-test arm (US$889.79) than in the control (US$753.17). Despite the low yield, the cost of identifying one HIV-positive person through our intervention was in the range of what previous community-based testing campaigns reported across sub-Sahara Africa (US$60.20 to US$1725.30) (7, 12, 14–16, 27–29) and lower than in the Zambian secondary self-test distribution trial (US$1,028.46) (19). The variability in cost estimates across the studies depends on the coverage achieved, the HIV prevalence, the intervention offered, and most importantly, the positivity rate.

Our study has several limitations. First, our micro-costing model did not capture all individual- and population-level costs and benefits of the intervention, and no quality-adjusted life years gained or disability-adjusted life years averted were included. Thus, these results should not be interpreted as a cost-effectiveness analysis. Second, the analysis is limited to a provider perspective which excluded key direct and indirect costs incurred by the clients when accessing testing services. However, the intervention offered self-testing at home and self-test return at the nearby village health worker, resulting in minimal time needed and low transport costs for the clients. Third, the study did not include a time and motion component, which would have given a more accurate reflection of the staff time involved for each activity.

## Conclusion

A self-testing strategy yielding high coverage and the optimal integration of the self-test follow-up in the existing health system resulted in a low incremental cost of secondary self-test distribution during home-based HIV testing in Lesotho. This secondary self-test distribution approach resulted in lower costs per person tested than standard home-based testing alone. These results may inform the current large-scale roll-out of HIV self-tests in Africa—also driven by the COVID-19 pandemic—and should be taken into account in home-based testing policies in similar settings.

## Data Availability Statement

The original contributions presented in the study are included in the article/Supplementary Material, further inquiries can be directed to the corresponding author/s.

## Author Contributions

LM built the micro-costing model with inputs from AA, NL and TG. AA drafted the first version of the manuscript. TG provided the HIV testing data from HOSENG trial. TL and MK provided costing source data. All authors read, critically revised, and approved the final manuscript. The corresponding author had full access to all the data in the study and had final responsibility for the decision to submit for publication.

## Conflict of Interest

The authors declare that the research was conducted in the absence of any commercial or financial relationships that could be construed as a potential conflict of interest.
