# Supplementary material for: Reaching Absent and Refusing Individuals During Home-Based HIV Testing Through Self-Testing—at What Cost?
Source: Front Med (Lausanne). 2021 Jun 29;8:653677. doi: 10.3389/fmed.2021.653677 (PMC8276095; doi:10.3389/fmed.2021.653677)
Supplement: Supplementary file 1 [file Data_Sheet_1.docx]

Supplementary Material

**Supplementary Figure 1a.** Testing Data and Scenarios in Control Arm

**Supplementary Figure 1b.** Testing Data and Scenarios in Intervention Arm

**Supplementary Table 1.** Description of cost items

| **ITEM** | **DESCRIPTION** | **INPUT** | **SOURCE** |
| --- | --- | --- | --- |
| **Trainings** | Three different trainings were involved: a) Training of the Campaign Team (Control & Intervention Arm), b) HIV testing and counselling refresher training for all VHWs (Control and Intervention Arm), c) Oral self-test training for VHWs (Intervention Arm only). | Per diem, lunch, transport, training material for trainees and trainers | Trial expenditure records |
| **Logistics** | The campaign was conducted with 3 Toyota Fortuner 4x4, year model 2016. Two cars were rented. One car was provided by the research organization and thus discounted and annualized. | Car rental, driver, maintenance, fuel | Trial expenditure records |
| **Clinic overhead** | Clinic overhead costs were calculated based on the size of the HIV testing and counselling space at an average health clinic in Lesotho | Water, electricity, waste & cleaning management | Lesotho Public Health Sector Expenditure Review 2017 |
| **Campaign equipment** | Campaign equipment included personal equipment for the campaign counselors such as safety boxes, bags stationary and lunches | Material costs | Trial expenditure records |
| **Consumables** | HIV blood-based tests (Determine HIV1-2 & UniGold HIV1-2), HIV oral-fluid self-tests (OraQuick), gloves, fingerprick at both the community and facility level | Material costs. HIV blood-based tests were partly donated by MoH as part of the collaboration, thus they were valuated | Trial expenditure records |
| **Headquarter-based staff** | Campaign organizers, 1 per district, coordinating the campaign from the office, compiling data and ensuring consumable supply | Salary costs for 5 months | Trial expenditure records |
| **Field- and clinic-based staff** | Campaign counsellors conducting the HIV testing campaign. Clinic HIV testing counselors and village health workers from Ministry of Health, performing the follow-up of distributed self-tests | Personnel time; Staff cost per minute based on the monthly salary | Trial expenditure records (Campaign counsellors); Ministry of the Public Service Circular Notice No. 10 of 2017 (Clinic counsellors and village health workers; Staff interviews (personnel time) |
